# Hidden in the dark: A new record and species of cave-dwelling *Palaumysis* (Crustacea, Mysidacea) from Sipadan Turtle Tomb, Malaysia, with identification keys to world species

**DOI:** 10.3897/zookeys.1284.192633

**Published:** 2026-07-08

**Authors:** Abdul-Rahim Azman, Shozo Sawamoto, Abu-Bakar Ahmad-Zaki

**Affiliations:** 1 Department of Earth Sciences and Environment, Faculty of Science and Technology, Universiti Kebangsaan Malaysia, 43600 UKM Bangi, Selangor, Malaysia Department of Earth Sciences and Environment, Faculty of Science and Technology, Universiti Kebangsaan Malaysia Bangi Malaysia; 2 20-21 Tsukimi-cho, Shimizu, Shizuoka 424-0853 Japan Unaffiliated Shimizu Japan; 3 Jetama Sdn Bhd, Unit No B2-10-03, Block B, The Riverson Suites, Coastal Road, 88100 Kota Kinabalu, Sabah, Malaysia Jetama Sdn Bhd, Unit No B2-10-03 Kota Kinabalu Malaysia

**Keywords:** Malaysia, marine cave, Mysidae, new species, Palaumysinae, *Palaumysis jetama*, Sipadan, systematics

## Abstract

A new species of the cave-dwelling mysid genus *Palaumysis* Bacescu & Iliffe, 1986 is described from the marine cave Turtle Tomb, Sipadan Island, Sabah, Malaysia. This represents the first record of the genus from Malaysian waters. The new species belongs to the group of *Palaumysis* species characterized by a reduced carapace, exposing the last five thoracic somites, but is distinguished from its congeners by the combination of adult body size exceeding 2.5 mm, a 5-segmented inner flagellum of the antennule, and the absence of fine hairs on the male fourth pleopod. *Palaumysis jetama***sp. nov**. is currently known only from the type locality. A revised identification key to all currently known species of Palaumysinae is presented.

## Introduction

Sipadan Island, lying within the Coral Triangle and separated from the Borneo continental shelf by a trench reaching depths of over 600 m, is globally celebrated for its rich marine biodiversity. The island first captured worldwide attention through Jacques Cousteau’s documentary Ghost of the Sea Turtles, which portrayed its remarkable underwater ecosystems and cemented its reputation as one of the finest dive sites in the world (Wood 1994). Despite this recognition and ecological importance, the biodiversity of Sipadan remains surprisingly underdocumented. Current records indicate only four endemic species from this unique locality: three copepods, *Sipadania celerinae* Humes & Lane, 1993, *Sipadantonius roihani* Boonyanusith, Wongkamhaeng & Azman, 2024, and *Peltidium penyu* Boonyanusith, Wongkamhaeng & Azman, 2026, along with one amphipod, *Talorchestia sipadan* Lowry, Springthorpe & Azman, 2017.

Among the many understudied crustacean groups in this region are mysids (order Mysida), for which research in Sabah waters remains extremely limited. Despite this, mysids are considered one of the most abundant and widely distributed crustaceans worldwide, inhabiting a wide range of aquatic environments but occurring predominantly in marine habitats (Gan et al. 2010; Wittmann et al. 2014; Meland et al. 2015). To date, only one species, *Idiomysis bumbumiensis* Nurshazwan, Sawamoto & Rahim, 2021, has been recorded from the east coast waters of Sabah, and no mysid species of the genus *Palaumysis* have been reported from this region.

One of the most distinctive features of this renowned island is its submerged marine cave system, commonly referred to as the “Turtle Tomb.” This extensive labyrinth extends for approximately 400 meters and is made up of complex limestone formations. The main entrance to the cave lies at a depth of around 20 meters, providing access to a convoluted network of chambers and passages that have become an iconic yet challenging site for scientific exploration and technical diving. The Turtle Tomb marine cave system represents a distinctive, yet still poorly documented habitat within Sipadan. Given the limited study of mysids in Sabah waters and the absence of previous records of *Palaumysis* from the region, the material collected from this cave is of particular taxonomic interest. Here we describe a new species of *Palaumysis* from Turtle Tomb and provide an updated key to the known species of the genus.

## Material and methods

**Figure 1.**
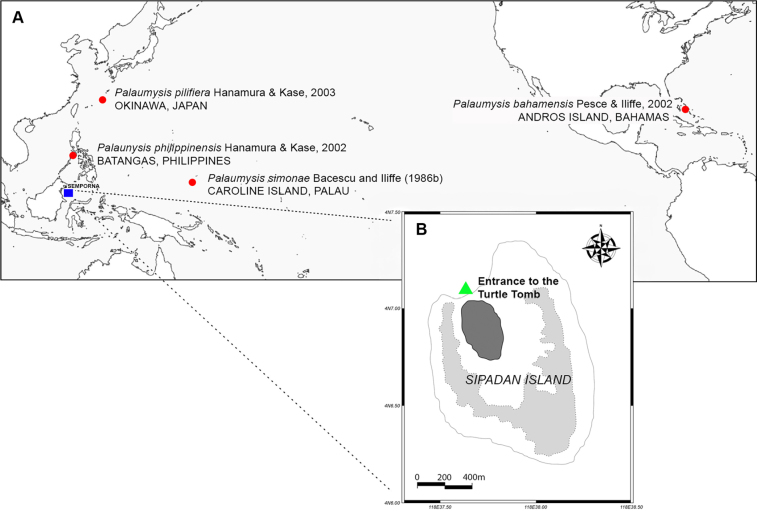
**A**. Map showing the geographic locations of the type localities of previously described *Palaumysis* spp. (red dots) and of *Palaumysis jetama* sp. nov. (blue square); **B**. Map of Sipadan Island, showing the entrance to the Turtle Tomb cave (green triangle).

**Figure 2.**
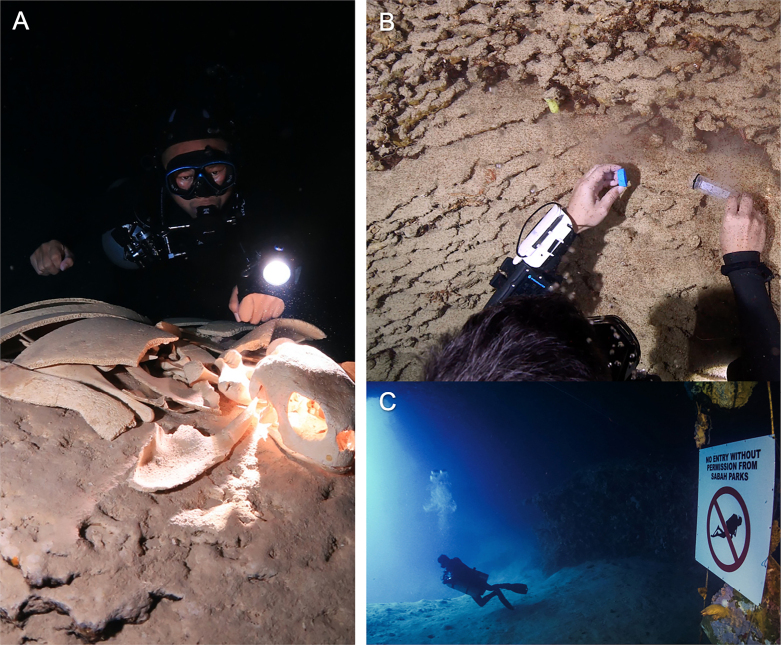
**A**. Second author assessing skeletal remains in the Sipadan Turtle Tomb; **B**. First author collecting mysid specimens using a 50 ml Polypropylene tube; **C**. View from the entrance of the cave showing a restricted access sign.

Specimens were collected in the Turtle Tomb, Sipadan Island, Sabah, Malaysia (4°07'04.8"N, 118°37'41.0"E) on 9 September 2025 (Fig. 1). Collections were made manually (Fig. 2B) with a 50 ml polypropylene conical centrifuge tube from the water column at 20–24 m depth. Living specimens were photographed. Specimens used for morphological descriptions were preserved in 10% formaldehyde before examination and subsequently preserved with 85% ethyl alcohol after sorting in the laboratory. The body length (**BL**; mm) was measured from the anterodorsal end of the carapace to the apex of the telson. The type material was dissected using a stereomicroscope (Olympus SZX9) and mounted on a temporary slide with a glycerol-ethanol mixture for illustrative purposes. Structures were examined and sketched under an optical microscope (Olympus BX43), equipped with a camera lucida. They were then ‘inked’ and scanned into Adobe Illustrator CS6 following the methods of Coleman (2003). Materials are deposited at the Universiti Kebangsaan Malaysia Muzium Zoologi (**UKMMZ**), Malaysia, National Museum of Nature and Science (**NSMT**), Japan and the Sabah Parks Zoological Collection in Semporna, Sabah (in accordance with the requirements stated in the Sabah Biodiversity Council – License Ref. No. JKM/MBS.1000-2/2/1 JLD.1 [223]).

## Systematics

### Order Mysida Haworth, 1825

**Family Mysidae Haworth, 1825**

**Subfamily Palaumysinae Wittman, 2013**

#### Genus Palaumysis

Taxon classificationAnimaliaMysidaMysidae

Bacescu & Iliffe, 1986

D3163CAE-4EDC-51B4-BD52-FF66CFE8AD4F

##### Type species.

*Palaumysis simonae* Bacescu & Iliffe, 1986.

##### Diagnosis.

See Hanamura and Kase (2002).

##### Species composition.

*Palaumysis* contains five (including the new species): *Palaumysis bahamensis* Pesce & Iliffe, 2002; *P. jetama* sp. nov.; *P. philippinensis* Hanamura & Kase, 2002; *P. pilifera* Hanamura & Kase, 2003; *P. simonae* Bacescu & Iliffe, 1986.

#### Palaumysis jetama sp. nov.

Taxon classificationAnimaliaMysidaMysidae

E554878B-BDF7-5D31-98E8-CF69A8E9892C

https://zoobank.org/28A27A9F-F8B0-4491-A93A-4FA84EB19F31

Figs 3, 4, 5

##### Type material.

**Holotype**, • adult male (BL. 2.52 mm, UKMMZ-1623); **Allotype**, • ovigerous female (BL. 2.88 mm, UKMMZ-1624); **Paratypes**, • seven males (BL. 2.52–2.70 mm), seven females (BL. 2.53–2.65 mm) (UKMMZ-1625); five females (BL. 2.61–2.85 mm, UKMMZ-1626); ten males, five females (NSMT-Cr 33264), Turtle Tomb, Sipadan Island, Sabah, Malaysia, 4°07'04.8"N, 118°37'41.0"E, SCUBA diving, 9 September 2025, 22 m depth, collectors: Azman B.A.R. and Ahmad-Zaki A.B.

##### Type locality.

Turtle Tomb of Sipadan Island, Sabah, Malaysia (4°07'04.8"N, 118°37'41.0"E), manual collection using 50 ml polypropylene conical centrifuge tube, depth ~ 20 m.

##### Diagnosis.

Adult body length not exceeding 3 mm. Carapace reduced, leaving the last five thoracic somites exposed. Eyes well developed, with globular cornea. Antennular peduncle with third segment robust and longest; base of outer flagellum bearing three to five sensory setae. Inner flagellum very short, composed of two to five articles; male lobe undeveloped. Antennal scale vestigial to rudimentary. Labrum evenly convex anteriorly, lacking frontal process or spine. Thoracic endopods 3–8 rather stout; carpo-propodi unsegmented and shorter than meri. Male pleopods rudimentary and unsegmented; pleopod 1 bearing a long procurved distal seta, pleopod 4 terminating in a strong spine. Female pleopods tiny and unsegmented. Marsupium composed of two pairs of rudimentary oostegites. Telson entire, subtriangular, armed apically with a pair of articulated or non-articulated spines, and lacking plumose setae on all margins. Exopod of uropod shorter than endopod, armed laterally with a few setae on posterior half.

##### Description.

**Figure 3.**
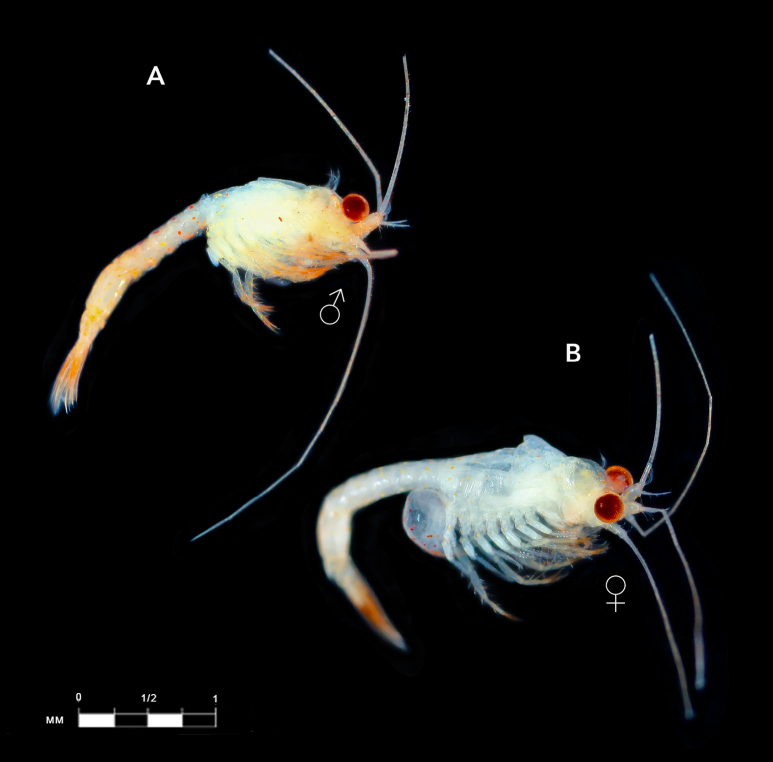
*Palaumysis jetama* sp. nov. (freshly fixed). **A**. Lateral view of the holotype (BL. 2.52 mm, UKMMZ-1632); **B**. Lateral view of allotype (BL. 2.88 mm, UKMMZ-1624), Turtle Tomb, Sipadan Island, Sabah, Malaysia.

**Figure 4.**
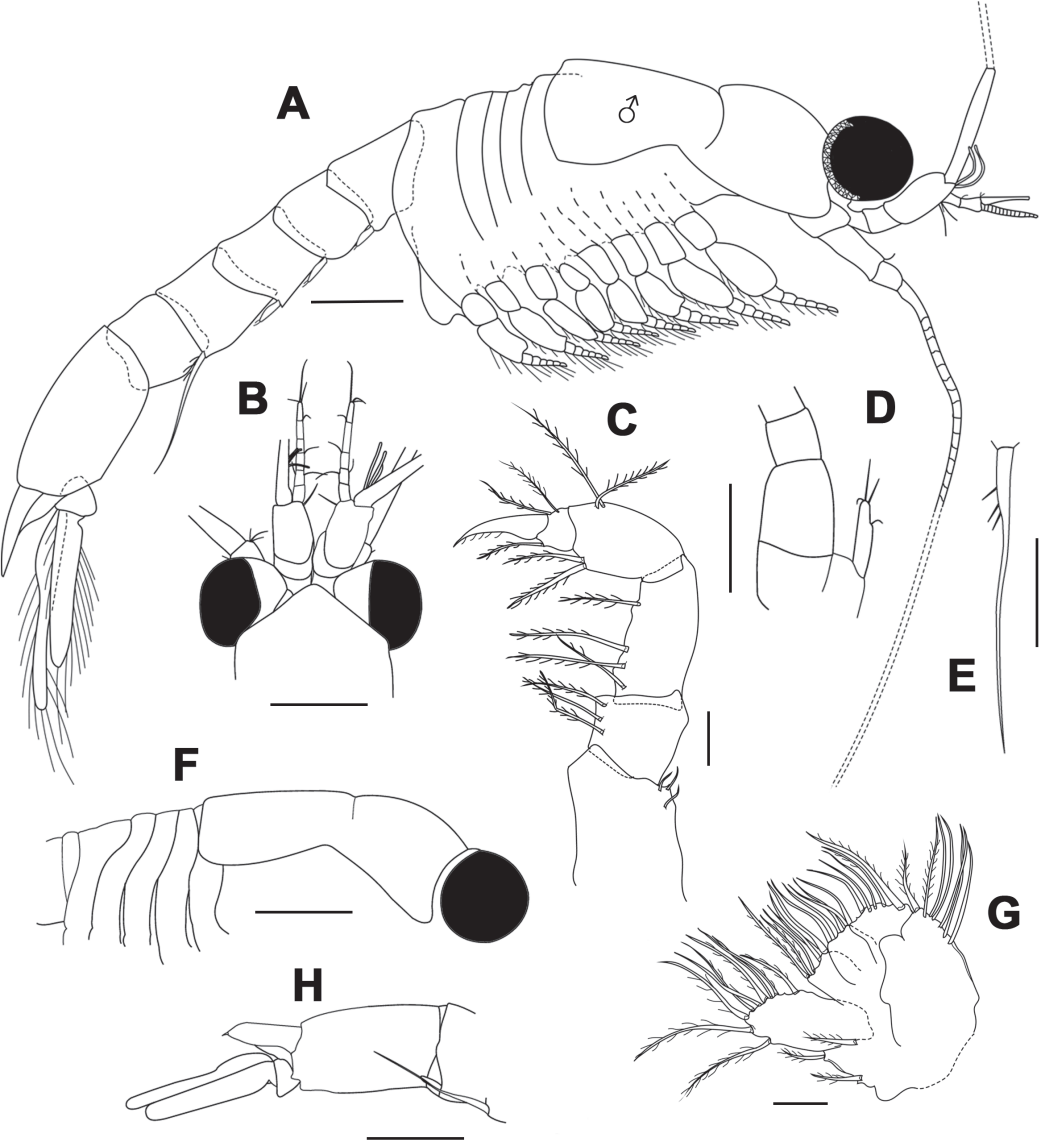
*Palaumysis jetama* sp. nov., holotype, male, 2.52 mm, UKMMZ-1632. Turtle Tomb, Sipadan Island, Sabah, Malaysia. **A**. Habitus, male; **B**. Dorsal view of anterior part of cephalon; **C**. Right 1^st^ thoracic endopod, external; **D**. Ventral view of left antenna; **E**. Right 4^th^ male pleopod; **F**. Lateral view of thorax; **G**. Right maxilla; **H**. Lateral view of posterior end of abdomen. Scale bars: 0.1 mm (**C, D, E, G**); 0.2 mm (**B, F, H**); 0.25 mm (**A**).

**Male. *Head and cephalic appendage***. Body pale white and translucent with reddish body parts (Fig. 3); cornea bright orange to brownish-red (Fig. 3). Carapace (Fig. 4A, F) reduced in size, dorsal and lateral carapace folds weakly developed; frontal margin anteriorly produced into a subtriangular rostrum with a rounded apex; lateral margin of rostrum uniformly concave; posterior margin truncate, exposing five or six thoracic somites in dorsally; posterolateral part of the carapace with a narrow but distinct free lobe, resulting in a subquadrate posteroventral corner; cervical sulcus distinct; and anterolateral corner of the carapace acutely pointed (Fig. 4B).

**Figure 5.**
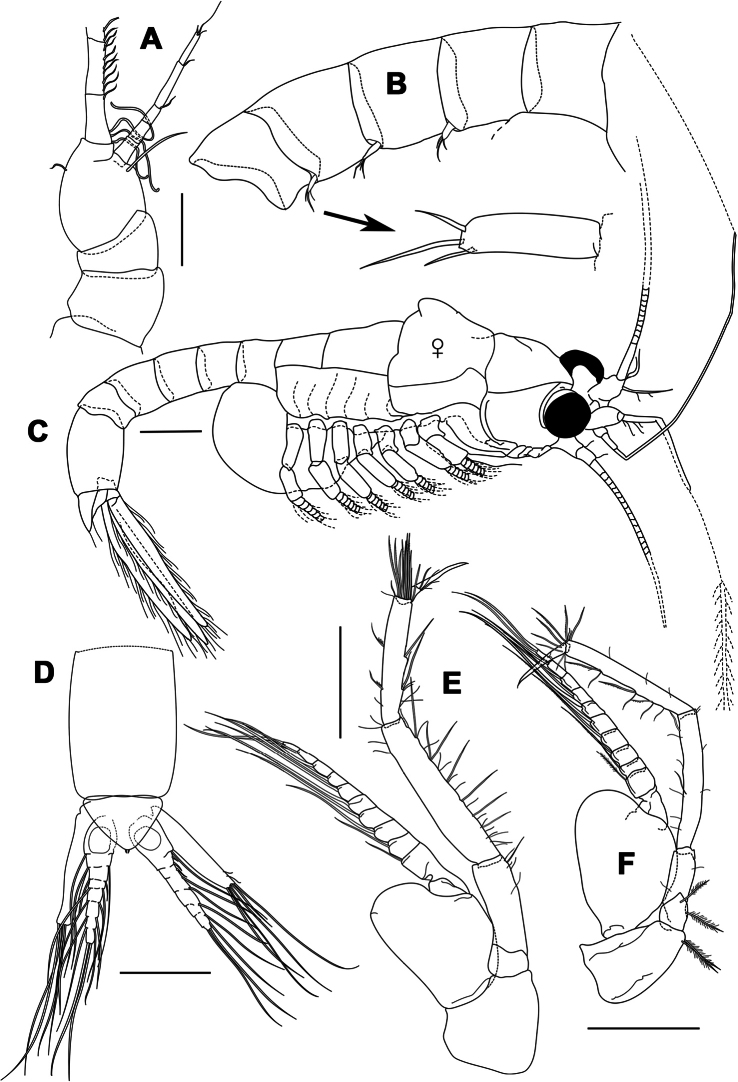
*Palaumysis jetama* sp. nov., paratype, female, 2.88 mm, UKMMZ-1624, Turtle Tomb, Sipadan Island, Sabah, Malaysia. **A**. Dorso-lateral view of left antennule; **B**. Female abdominal somites with enlarged 4^th^ pleopod; **C**. Habitus, female; **D**. Dorsal view of 6^th^ abdominal somite, telon and uropods; **E**. Left 3^rd^ thoracic limb 2; **F**. Left 8^th^ thoracic limb 6. Scale bars: 0.1 mm (**A, B, E, F**); 0.2 mm (**D**); 0.25 mm (**C**).

***Eye*** (Fig. 4A, B, F) large, cornea globular, well pigmented. Antennule peduncle (Fig. 5A) with three segments; the basal segment is the longest, about 1.3 times as long as the median segment along mesial margin, unarmed; the median segment is the shortest with a ventral short lobe on subterminal position, unarmed; the terminal segment is slightly shorter than the first/basal segment, armed with long seta at distomesial corner and short seta at distolateral corner; outer flagellum long, bearing 10 sensory setae at proximal inner part; inner flagellum short, 5-segmented. Antenna (Fig. 4D) scale rudimentary, with three apical setae (two medium size and one short) and one short seta along lateral margin. Maxilla (Fig. 4G) as illustrated. First thoracic endopod as illustrated (Fig. 4C). Thoracic limbs well developed and similar in shape. Second thoracic endopod with robust basis; subquadrate preischium; ischium is more than half the length of merus, armed with fine setae; merus longest with setae along outer margin; carpopropodus slightly shorter than merus with setae along both margins; dactylus is more than half the length of carpopropodus with long and mid-length setae distally (Fig. 5E). Sixth thoracic endopod generally similar in shape with 2^nd^ thoracic but with plumose setae on outer margins of basis and preischium; preischium triangular; and ischium is half the length of merus (Fig. 5F).

***Pleon*** (Fig. 4A, H) slightly depressed dorsoventrally; first to fifth somites gradually increasing in length posteriorly, sixth somite longest, about 2 times as long as fifth one.

***Pleopods in male*** (Fig. 4A) second, third and fifth pleopods similar in general shape; fourth pleopod (Fig. 4E) terminating into strong spine, extending beyond midlength of sixth somite, bearing rows of short setules near midlength, articulation between pleopodal plate and terminal spine indistinct.

***Uropod*** (Figs 4H, 5D) extending distinctly beyond end of telson. Exopod of uropod narrow, truncate distally, lateral margin without seta, distal margins armed with numerous long setae (Fig. 5D). Endopod of uropod narrow, distinctly longer than exopod, armed with numerous long setae on lateral and mesial margins; sutures strongly developed, giving an almost segmented appearance; statocyst present (Fig. 5D). Telson (Fig. 5D) subtriangular, width distinctly longer than length, armed with two distal spines.

**Female**. Similar to male, except for the following differences: body stouter and more robust in lateral view (Fig. 3), due to the presence of a well-developed marsupium; thoracic region deeper and broader than in male; brood pouch conspicuous, occupying much of the ventrolateral thoracic region. Antennules, antennae, eyes, and abdominal somites generally as in male; pleopod 4 (Fig. 5B) rudimentary, short, unsegmented, bearing three to four short distal setae; not extending beyond the posterior margin of the somite.

##### Etymology.

The species epithet *jetama* is dedicated to Jetama Sdn. Bhd., in recognition of the company’s support and contribution to this research. Their funding was instrumental in facilitating the fieldwork component of the study and enabling the discovery and documentation of this species.

##### Distribution.

This species is known only from Turtle Tomb of Sipadan Island, Sabah, Malaysia.

##### Remarks.

*Palaumysis jetama* sp. nov., is readily distinguished from all currently known congeners by a unique combination of body size and antennular morphology. Adults exceed 2.5 mm in total length, placing the species among the larger members of the genus. Within this size class, *P. jetama* sp. nov. differs clearly from *P. bahamensis* Pesce & Iliffe, 2002 in possessing a 5-segmented inner flagellum of the antennule rather than a 2-segmented flagellum with an extremely elongate distal article.

The new species further differs from the smaller congeners *P. simonae* Băcescu & Iliffe, 1986, *P. pilifera* Hanamura & Kase, 2003, and *P. philippinensis* Hanamura & Kase, 2002 by its larger adult size (> 2.5 mm versus < 2.5 mm). It is additionally separable from *P. pilifera* by the absence of fine hairs on the male fourth pleopod in *P. jetama* sp. nov. *Palaumysis simonae* differs in having a naked antennal scale, whereas *P. philippinensis* bears a terminal seta and several short lateral setae on the antennal scale, characters not observed in the new species.

Among the described congeners, *Palaumysis jetama* sp. nov. most closely resembles *P. philippinensis*. However, adult specimens of the new species differ from *P. philippinensis* by at least three characters: the length ratio of segments 1 and 2 of antennal scale (2:1 in *P. jetama* versus 1:4 in *P. philippinensis*); the thoracic exopods have more than 10 segments in *P. jetama* versus fewer than 7 in *P. philippinensis*); and the telson is broader than long in *P. jetama* versus longer than broad in *P. philippinensis*.

The discovery of *P. jetama* sp. nov. extends the known distribution of *Palaumysis* to Malaysian Borneo and adds a new record for the central Indo-West Pacific. With species now known from Palau, Japan, the Philippines, the Bahamas, and Malaysia, the genus shows a disjunct circumtropical distribution associated with marine cave habitats. This pattern is consistent with the broader biogeographic significance of marine caves as refugia for relictual crustacean lineages, although the historical processes underlying the present distribution of *Palaumysis* remain to be tested.

## Discussion

Observations in Turtle Tomb indicate that the cave supports a dense and persistent aggregation of zooplankton, particularly within 10–20 m of the entrance, and *Palaumysis jetama* sp. nov. appeared to be the visually dominant component of that aggregation. Although quantitative ecological data are not yet available, this repeated field observation suggests that the species may represent an important component of the local cave community and warrants further study of its abundance, trophic role, and seasonal persistence.

The five congeneric species exhibit notable differences in their recorded depth distributions, although all are restricted to the marine cave habitat (Table 1). *Palaumysis jetama* sp. nov. was collected at the shallowest depth among other *Palaumysis* species, around 20 m, distinguishing it from its congeners, which typically occur at greater depths. *Palaumysis simonae* was found at 36 m, *P. pilifera* at 40 m, *P. philippinensis* at 46 m, and *P. bahamensis* at 60–70 m. Similarly, the cave-associated mysid *Gironomysis lalanai* from the anchialine cave system of Playa Girón, Cuba, was reported from approximately 15 m depth, indicating that some subterranean mysids may persist within relatively shallow cave environments. The comparatively shallow occurrence of *P. jetama* sp. nov. and *G. lalanai* may reflect stronger marine connectivity, partial light penetration near cave entrances, or differing ecological tolerances compared with deeper-dwelling cave mysids. These depth differences may reflect ecological partitioning among species, but broader sampling across cave systems and depth zones will be required before such a pattern can be evaluated confidently.

**Table 1.** Distributional records, habitat depths (m), and references of known species of *Palaumysis*, including the newly described *P. jetama* sp. nov.

**Species**	**Geographic location**	**Depth recorded (m)**	**Reference**
*P. simonae*	Caroline Island, PALAU	36	Bacescu and Iliffe (1986)
*P. pilifera*	‘Daidokutsu’ cave, Ieshima Island, Okinawa, JAPAN	40	Hanamura and Kase (2003)
*P. philippinensis*	Mapating Cave, Maricaban Island, Batangas, PHILIPPINES	46	Hanamura and Kase (2002)
*P. bahamensis*	Atlantis Blue Hole, South Andros Island, the BAHAMAS	60–70	Pesce and Iliffe (2002)
*P. jetama* sp. nov	Turtle Tomb, Sipadan Island, MALAYSIA	~20	This study

### Key to world species of Palaumysinae

**Table:** 

1	Carapace covering nearly the entire thorax	***Gironomysis lalanai***
–	Carapace leaving posterior thoracomeres exposed	**2**
2	Adults less than 2.5 mm in body length	**3**
–	Adults more than 2.5 mm in body length	**5**
3	Male fourth pleopod without setae throughout its length	**4**
–	Male fourth pleopod with rows of fine hairs near mid-length	***Palaumysis pilifera***
4	Antennal scale without setae	***Palaumysis simonae***
–	Antennal scale with a long terminal seta and several short setae along the lateral margin	***Palaumysis philippinensis***
5	Inner flagellum of antennule 2-segmented, distal segment extremely elongated	***Palaumysis bahamensis***
–	Inner flagellum of antennule 5-segmented	***Palaumysis jetama* sp. nov**.

## Supplementary Material

XML Treatment for Palaumysis

XML Treatment for Palaumysis jetama

## Additional information

### Artificial Intelligence (AI) use

The authors accept full responsibility for the content of the manuscript, including the disclosure of any use of AI.

No AI tools were used in the preparation of this manuscript.

### Author ORCIDs

A.-R. Azman https://orcid.org/0000-0002-8964-6638

S. Sawamoto https://orcid.org/0000-0002-5898-4917

A.-B. Ahmad-Zaki https://orcid.org/0009-0006-0402-8774

### Data availability

All of the data that support the findings of this study are available in the main text.
